# Phylogeography and Genetic Diversity of *Francisella tularensis* subsp. *holarctica* in France (1947–2018)

**DOI:** 10.3389/fmicb.2020.00287

**Published:** 2020-03-04

**Authors:** Maëllys Kevin, Guillaume Girault, Yvan Caspar, Moulay Ali Cherfa, Christiane Mendy, Herbert Tomaso, Dolores Gavier-Widen, Raquel Escudero, Max Maurin, Benoît Durand, Claire Ponsart, Nora Madani

**Affiliations:** ^1^Paris-Est University/ANSES, Animal Health Laboratory, Maisons-Alfort, France; ^2^Laboratoire de Bactériologie-Hygiène Hospitalière, Centre National de Référence des Francisella, CHU-Grenoble-Alpes, Grenoble, France; ^3^Friedrich-Loeffler-Institut, Jena, Germany; ^4^Department of Pathology and Wildlife Diseases, National Veterinary Institute, Uppsala, Sweden; ^5^Centro Nacional de Microbiología, Instituto de Salud Carlos III, Madrid, Spain

**Keywords:** canonical single nucleotide polymorphism (canSNP), genotyping, molecular epidemiology, tularemia, whole-genome sequencing (WGS)

## Abstract

In France, tularemia is caused by *Francisella tularensis* subsp. *holarctica* and is a sporadic disease affecting mainly wildlife animals and humans. *F. tularensis* species presents low genetic diversity that remains poorly described in France, as only a few genomes of isolates from the country are available so far. The objective of this study was to characterize the genetic diversity of *F. tularensis* in France and describe the phylogenetic distribution of isolates through whole-genome sequencing and molecular typing. Whole genomes of 350 strains of human or animal origin, collected from 1947 to 2018 in France and neighboring countries, were sequenced. A preliminary classification using the established canonical single nucleotide polymorphism (canSNP) nomenclature was performed. All isolates from France (except four) belonged to clade B.44, previously described in Western Europe. To increase the resolution power, a whole-genome SNP analysis was carried out. We were able to accurately reconstruct the population structure according to the global phylogenetic framework, and highlight numerous novel subclades. Whole-genome SNP analysis identified 87 new canSNPs specific to these subclades, among which 82 belonged to clade B.44. Identifying genomic features that are specific to sublineages is highly relevant in epidemiology and public health. We highlighted a large number of clusters among a single clade (B.44), which shows for the first time some genetic diversity among *F. tularensis* isolates from France, and the star phylogeny observed in clade B.44-subclades revealed that *F. tularensis* biodiversity in the country is relatively recent and resulted from clonal expansion of a single population. No association between clades and hosts or clinical forms of the disease was detected, but spatiotemporal clusters were identified for the first time in France. This is consistent with the hypothesis of persistence of *F. tularensis* strains found in Western Europe in the environment, associated with slow replication rates. Moreover, the presence of identical genotypes across long periods of time, and across long distances, supports this hypothesis but also suggests long-distance dispersal of the bacterium.

## Introduction

Tularemia is a zoonosis caused by *Francisella tularensis*, a Gram-negative facultative intracellular bacterium (Sjöstedt, 2007). Only two subspecies of *F. tularensis* are causative agents of tularemia: *F. tularensis* subsp. *tularensis* (*Ftt*, also referred to as type A) in North America, and *F. tularensis* subsp. *holarctica* (*Fth*, type B), classically in the whole northern hemisphere, but recently detected in Australia (Eden et al., 2017). In France, the first human case was reported in 1945 (Girard et al., 1946), and the bacterium was first isolated from hares in 1947 (Paille, 1950). Tularemia is endemic in France, where the incidence of this zoonosis has constantly been increasing over the past two decades, reaching 50–100 human cases per year (Santé Publique France, 2019). However, very few studies have investigated the phylogeography and genetic diversity of *F. tularensis* isolates from France (Vogler et al., 2009, 2011; Dwibedi et al., 2016), and little is known about the epidemiology of tularemia in France.

*Francisella tularensis* is a highly infectious microorganism both in humans and in many animal species (Ellis et al., 2002). This bacterium is considered a category A potential biological threat agent, as classified by the CDC (Centers for Disease Control and Prevention, Atlanta, GA, United States). The most virulent *Ftt* strains may induce severe pneumonia, with a fatality rate of up to 30% (Maurin and Gyuranecz, 2016). Six clinical forms of tularemia are classically recognized in humans, depending on the route of infection with *F. tularensis*. Patients usually present with regional lymphadenopathy that can be isolated (glandular form) or combined with a skin ulcer (ulceroglandular form), conjunctivitis (oculoglandular form), or pharyngitis (oropharyngeal form). The two remaining clinical forms are systemic diseases, i.e. the pneumonic form and the typhoidal form.

Tularemia is a notifiable disease in most endemic countries, including the United States (USA) and France, in human health as well as in animal health. Small rodents and lagomorphs are considered to be key reservoirs and amplification hosts of *F. tularensis*, and a primary source of human infections (Gyuranecz et al., 2012; Maurin and Gyuranecz, 2016). Arthropods (especially ticks) and the environment may also be a long-term reservoir of *F. tularensis*. In Europe, two life cycles (terrestrial and aquatic) have been described for *Fth* strains (Maurin and Gyuranecz, 2016; Luque-Larena et al., 2017). Interestingly, two different life cycles have been described in the USA for *Ftt* and *Fth* strains, with only the latter strains associated with aquatic rodents such as beavers, muskrats and voles (Kugeler et al., 2009). However, many aspects of the epidemiology of tularemia remain poorly characterized. In particular, the true reservoirs and the modes of long-term survival and spread of *F. tularensis* remain to be fully characterized.

**FIGURE 1:**
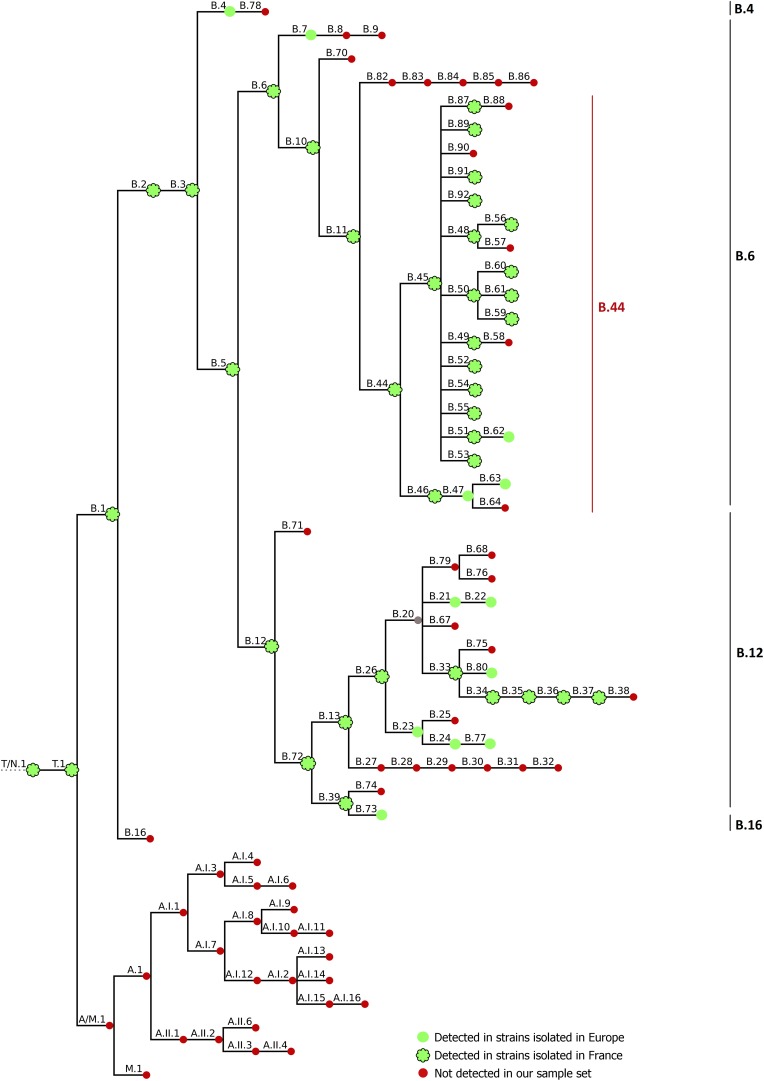
CanSNP nomenclature generated by CanSNPer (Lärkeryd et al., 2014). Clades detected in strains isolated in Europe are indicated with green circles; strains detected in strains isolated in France are contoured in black, and clades that were not detected in our sample set are indicated in red.

Recent improvements in our knowledge of the epidemiology of tularemia have been achieved by the development of genotyping methods. Considering the clonal nature of the pathogen (Johansson and Petersen, 2010), only molecular methods with high discriminatory power allow us to distinguish closely related subpopulations at the strain level. As such, genotyping of *F. tularensis* strains is now mostly based on the identification of canSNP at a whole-genome scale, as a result of significant reductions in sequencing costs. This technique can be used to investigate deep phylogenetic relationships and enables good discrimination of bacterial strains (Keim et al., 2004). Four main clades have been described for *Fth*: B.4, B.6, B.12, and B.16 (Vogler et al., 2009) (Figure 1). Clade B.4 has been described mostly in North America, but has also been isolated in Norway, Sweden, and Germany (Vogler et al., 2009; Müller et al., 2013). In Europe, most strains belong to basal clades B.6 and B.12. Clade B.6 is found in Western Europe and North America, whereas clade B.12 has been isolated mainly in Eastern Europe, and groups together erythromycin-resistant strains (Vogler et al., 2009). In Germany, Switzerland, Sweden, Norway and Finland, both of these major clades have been detected (Karlsson et al., 2013; Müller et al., 2013; Origgi et al., 2014; Afset et al., 2015; Sissonen et al., 2015; Wittwer et al., 2018). Clade B.16 correlates with biovar *japonica*, isolated in Japan and recently in Turkey (Ulu-Kilic et al., 2013), China (Wang et al., 2014), and Australia (Eden et al., 2017).

In Europe, tularemia has been reported only since the 1930s, and Eurasian *Fth* isolates are more closely related than *Fth* isolates from the United States, which suggests the spread of *Fth* strains from the United States to Eurasia (Johansson et al., 2004). Also, it has been hypothesized that the current population of *Fth* in Western Europe expanded clonally from a founder population that originated near the Eastern border, resulting in a gradual reduction in genetic diversity from Eastern to Western Europe (Dwibedi et al., 2016). Recent phylogeographic studies based on genetic relationships between *F. tularensis* isolates supported the hypothesis of long-range dispersal of this bacterium over time, associated with slow replication rates and long-term persistence in the environment (Keim and Wagner, 2009; Dwibedi et al., 2016). Only clade B.Br.FTNF002-00, which correlates with clade B.11 (Vogler et al., 2009), a subclade of clade B.6, was described as the major clade in France in 2011 (Vogler et al., 2011). This study involving 103 isolates confirmed the hypothesis of the colonization of Western Europe by a single clone and emphasized the need for data on SNP within the B.11 clade. Further studies confirmed that isolates from France belong to a single subclade of clade B.11: B.45, among closely related subclades B.49, B.60 (subclade of B.50), B.54 and B.55 (Dwibedi et al., 2016).

The aim of this study was to characterize the biodiversity of *F. tularensis* in France and neighboring countries through whole-genome sequencing and genotyping of 350 strains of human and animal origin. The identification of specific and discriminative genetic markers and the investigation of phylogenetic relationships between isolates provided new knowledge about the epidemiology of tularemia in France. Obtaining the highest possible resolution was necessary to establish links between French *Fth* isolates, and potentially trace back the origin and history of contamination in a defined geographical area.

## Materials and Methods

### Bacterial Strains

A total of 350 *Francisella* strains were used in this study, including three reference strains (*F. novicida* ATCC 15482; *F. tularensis* subsp. *tularensis* ATCC 6223; and *F. tularensis* subsp. *holarctica* Live Vaccine Strain, ATCC 29684) and 347 European isolates of *F. tularensis* subsp. *holarctica* (Supplementary Table S1). These field strains were collected mostly in France (*n* = 304), from 1947 to 2018. Other European strains isolated in Germany (*n* = 19), Sweden (*n* = 19), Spain (*n* = 4), and Belgium (*n* = 1) were also characterized. Strains were selected as diversely as possible in terms of geographic and temporal distribution to account for biodiversity in France and to be able to place them in a global phylogenetic context with the other European strains. Field strains were isolated from different host species (Supplementary Table S1): mostly wild hares (*Lepus* sp., *n* = 245), humans (*n* = 81, including 72 strains isolated from 2006 to 2018 from clinical cases in the collection of the French National Reference Center for *Francisella*), captive mammals (capybara – *Hydrochoerus hydrochaeris*, non-human primates – unknown species, *n* = 13), wild mammals other than hares (rabbit – *Oryctolagus cuniculus*, wild boar – *Sus scrofa*, roe deer – *Capreolus capreolus*, *n* = 6), and ticks (unknown species, *n* = 2). Wild animals were collected thanks to the SAGIR network, which is an epidemiological surveillance network for terrestrial wild birds and mammals in France and aims to control wildlife diseases by monitoring mortality events without presuming etiology. *F. tularensis* species was confirmed by phenotypic methods and real-time PCR (Versage et al., 2003), and *holarctica* subspecies was confirmed by PCR (Tomaso et al., 2007).

### DNA Extraction

Genomic DNA was obtained from bacteria grown at 37°C on Difco^TM^ Cystin Heart Agar (BD247100, France), or chocolate blood agar medium with PolyVitex (PVX, Biomerieux, Marcy l’Etoile, France). DNA was extracted and purified using QIAGEN^®^ Genomic-tip 100/G columns and QIAGEN^®^ Genomic DNA Buffer sets from suspension calibrated to 3 McFarland units according to the manufacturer’s recommendations. Purified DNA was quantified in a Qubit 2.0 Fluorometer (Thermo Fisher Scientific, Rodano, MI, Italy) using the Qubit dsDNA BR Assay Kit (Thermo Fisher Scientific), and the Qubit dsDNA HS Assay Kit (Thermo Fisher Scientific), following the manufacturer’s instructions.

### Whole-Genome Sequencing and Variant Analysis

In this study, whole-genome sequences of 193 strains were obtained from a HiSeq 2500 instrument (Illumina) at the Genoscreen sequencing platform (Genoscreen, Lille, France) using a Nextera XT DNA library prep kit (Illumina). For the remaining 157 strains, library preparations were performed using the same Nextera XT DNA library prep kit (Illumina), according to the manufacturer’s recommendations, and whole-genome sequencing was performed with a MiSeq instrument at ANSES (Bacterial Zoonosis Unit and IdentyPath platform, Laboratory for Food Safety, Maisons-Alfort, France). All strains were subjected to paired-end sequencing (2 × 250 bp) and deep coverage ranged from 67 to 551 (Supplementary Table S1), which allows for robust SNP detection. Data quality was assessed using FastQC v0.11.4 (Andrews^1^), and quality trimming was performed with Trimmomatic v0.32 (Bolger et al., 2014). Genomes were assembled *de novo* using SPAdes v3.12 (Bankevich et al., 2012). Genomic metrics were checked using QUAST v4.4 (Gurevich et al., 2013). Annotation files were generated with Prokka v1.13.3 (Seemann, 2014) using the Franco-Iberian *Fth* FTNF002-00 genome (NC_009749) as a reference. Abricate v0.8.10 (Seemann, 2019) was used in order to detect genes associated with antibiotic resistance using ARG-ANNOT (Gupta et al., 2014), CARD (Jia et al., 2017), EcOH (Ingle et al., 2015), NCBI Bacterial Antimicrobial resistance Reference Gene Database (Accession: PRJNA313047) and ResFinder (Zankari et al., 2012) databases, with a minimum identity of 50%. CanSNPer v1.0.8 (Lärkeryd et al., 2014) was used to assign the established canSNP nomenclature to the sequenced strains. The *Fth* FTNF002-00 genome (NC_009749) was used as a reference genome for mapping assembly. A CanSNPer tree was used to represent the established and the new canSNP nomenclature. SNP calling was performed with the iVarCall2 workflow (Felten et al., 2017) based on the HaplotypeCaller algorithm of GATK (McKenna et al., 2010). Variants were then annotated with SnpEff v4.2 (Cingolani et al., 2012) on the vcf file based on the FTNF002-00 genome annotation from NCBI.

### Whole-Genome Phylogenetic Analysis

Based on the file of concatenated SNPs generated by iVarCall2, maximum likelihood (ML) phylogenetic trees were created with IQTree (Nguyen et al., 2015), SH-aLRT (Anisimova et al., 2011) and UFBoot2 (Hoang et al., 2018) supports, set to 1000 replicates), after using Modelfinder (Kalyaanamoorthy et al., 2017) to determine the best model (TVM model in this study). The best-scoring ML tree was graphically represented with iTOL viewer (Supplementary Figure S1) (Letunic and Bork, 2016) and compared with the canSNP nomenclature to detect new subclades. The ‘phyloFixedVar’ script of FixedVarTools (Felten et al., 2017) was then used with the annotated vcf file from the variant calling analysis, and the nwk file from the best-scoring ML tree, in order to detect specific variants of subclades associated with their corresponding annotations. Only non-homoplastic SNPs (i.e. not found in any other strains of other clusters) were selected for the canSNP nomenclature. Newly identified canSNPs were named in agreement with this existing nomenclature (Lärkeryd et al., 2014). The text files containing the SNP positions and the tree topology were adjusted with the newly identified subtypes. CanSNPs identified in our study were arbitrarily classified from 1st to 8th order of discrimination (D1–D8), and their positions on genome FTNF002-00 are listed in Supplementary Table S2.

Genomes of the NCBI database (*n* = 248) that were available on the assembly summary file^2^ (Supplementary Table S3) were screened using the new canSNP nomenclature to validate the newly identified canSNPs.

### Statistical Analysis

The association between clade membership and epidemiological covariates (host species and geographical distribution) was analyzed for D4 clades containing at least 10 strains isolated in France (B.49, B.50, B.55, B.87, B.250, and B.251).

A spatiotemporal analysis was performed on the subset of strains isolated from hares in France between 1985 and 2018 (*n* = 204) since these animals are considered a relatively homogeneous host population. The association between geographic and genetic distances was assessed using Mantel’s test (1000 repetitions), based on the SNP distance matrix, and on the geographic distance matrix computed by assigning each strain the coordinates of the centroid of the French administrative area (department) of isolation (i.e. the 3rd level of NUTS [Nomenclature of Territorial Units for Statistics] in France). For each D4 clade containing at least 10 strains isolated from hares (to ensure the robustness of the statistical analysis), spatiotemporal clusters were identified using a Bernoulli model, with cases as the strains belonging to the considered clade, and controls those belonging to the other clades. The time and space resolution levels were the year and the department of isolation, respectively. Circular scanning windows were used. Purely spatial clusters were allowed, but purely temporal clusters were not, the maximum extent of a cluster being set to 50% of the strain population.

R software (R Core Team, 2019) was used for statistical analysis and Mantel’s tests were performed using the ade4 package (Dray and Dufour, 2007). Spatiotemporal cluster analysis was performed using SaTScan (2019) v9.6^3^ The SNP distance matrix was computed using iVarCall2 (Felten et al., 2017).

## Results

### *In silico* SNP Typing With Previously Published canSNPs

#### Strains From France

Draft genomes were genotyped using CanSNPer (Lärkeryd et al., 2014), which enabled us to classify the 350 different strains among the already described canSNPs (Figure 1). Among the strains from France, 98.68% (*n* = 300/304) were assigned to subclades of clade B.44 (one subclade of B.6, Table 1), except four isolates that belonged to clade B.12, and more particularly subclades B.13 and B.39 (Table 2). Concerning strains in the B.44 clade, 93.37% of the French strains (*n* = 296/304) belonged to clade B.45. In clade B.45, two subclades of B.50 (B.59 and B.61) as well as clades B.48, B.56, B.52, B.53, B.87, B.89, B.91, and B.92 (D4) were identified for the first time in France. Interestingly, around 40% of the French strains (*n* = 121/304) were not assigned to any further subclade, which reflects the lack of resolution for French isolates.

**TABLE 1:** CanSNPer typing results for clade B.6 of *Francisella tularensis* subsp. *holarctica*.

**Country**	**No. of strains**	**B.6**																	**D1**
		
		**B.44**																	**D2**
		
			B.44*	**B.46**	**B.45**														**D3**
			
						B.45*	**B.48**	**B.49**	**B.50**	**B.51**	**B.52**	**B.53**	**B.54**	**B.55**	**B.87**	**B.89**	**B.91**	**B.92**	**D4**
France	**304**	**300**	3	1	**296**	116	2	41	57	1	5	5	5	17	35	9	1	2	
Germany	**19**	**12**	0	1	**11**	0	0	2	7	1	0	0	0	0	1	0	0	0	
Sweden	**19**	**0**	0	0	**0**	0	0	0	0	0	0	0	0	0	0	0	0	0	
Spain	**4**	**4**	0	0	**4**	0	0	0	1	0	3	0	0	0	0	0	0	0	
Belgium	**1**	**1**	0	0	**1**	0	0	1	0	0	0	0	0	0	0	0	0	0	
Ref.	**3**	**0**	0	0	**0**	0	0	0	0	0	0	0	0	0	0	0	0	0	
**Total**	**350**	**317**	**3**	**2**	**312**	**116**	**2**	**44**	**65**	**2**	**8**	**5**	**5**	**17**	**36**	**9**	**1**	**2**	

Clades marked with a star (*) include strains that could not be further discriminated. “No.”: number of strains. Bold values correspond to the total number of strains (total, or in clades B.44 and B.45).

**TABLE 2:** CanSNPer typing results for clade B.12 of *Francisella tularensis* subsp. *holarctica*.

**Country**	**No. of strains**	**B.12**							**D1**
		
			**B.13**			**B.39**			**D2**
			
				B.13*	**B.26**		B.39*	**B.73**	**D3**
France	**304**	**4**	**3**	0	3	**1**	1	0	
Germany	**19**	**6**	**6**	0	6	**0**	0	0	
Sweden	**19**	**17**	**14**	1	13	**3**	1	2	
Spain	**4**	**0**	**0**	0	0	**0**	0	0	
Belgium	**1**	**0**	**0**	0	0	**0**	0	0	
Ref.	**3**	**1**	**1**	0	1	**0**	0	0	
**Total**	**350**	**28**	**24**	**1**	**23**	**4**	**2**	**2**	

Clades marked with a star (*) include strains that could not be further discriminated. “No.”: number of strains. Bold values correspond to the total number of strains (total, or in clades B.44 and B.45).

#### Other Strains

When including all the strains, 90.57% (*n* = 317/350) were assigned to clade B.44 and its subclades. Almost 90% of the total strains (*n* = 312/350) were assigned to clade B.45 (a subclade of B.44), but 33% (*n* = 116/350) were not assigned to any further subclade. The remaining B.44 strains were assigned to B.46 (*n* = 2, including one strain in subclade B.63), or to B.44 without any further resolution (*n* = 3). Strains from Germany in our panel mostly belonged to subclades of B.44 (*n* = 12/19 – among which eleven belonged to subclades of B.45), as well as other subclades of clades B.4 (*n* = 1) and B.12, but more specifically B.33, subclade of B.26 (*n* = 6). The strain from Belgium belonged to clade B.49 and strains from Spain in our panel (*n* = 4) belonged to clade B.52, except for one assigned to clade B.61 (subclade of B.50). Two strains from Sweden belonged to clade B.7, while the other seventeen belonged to subclades of clade B.12. As expected, the two reference strains *F. novicida* ATCC 15482 and *F. tularensis* subsp. *tularensis* ATCC 6223 belonged to clade T/N.1 and A.II.2, respectively.

### Whole-Genome Phylogenetic Analysis and Definition of New canSNPs

**FIGURE 2:**
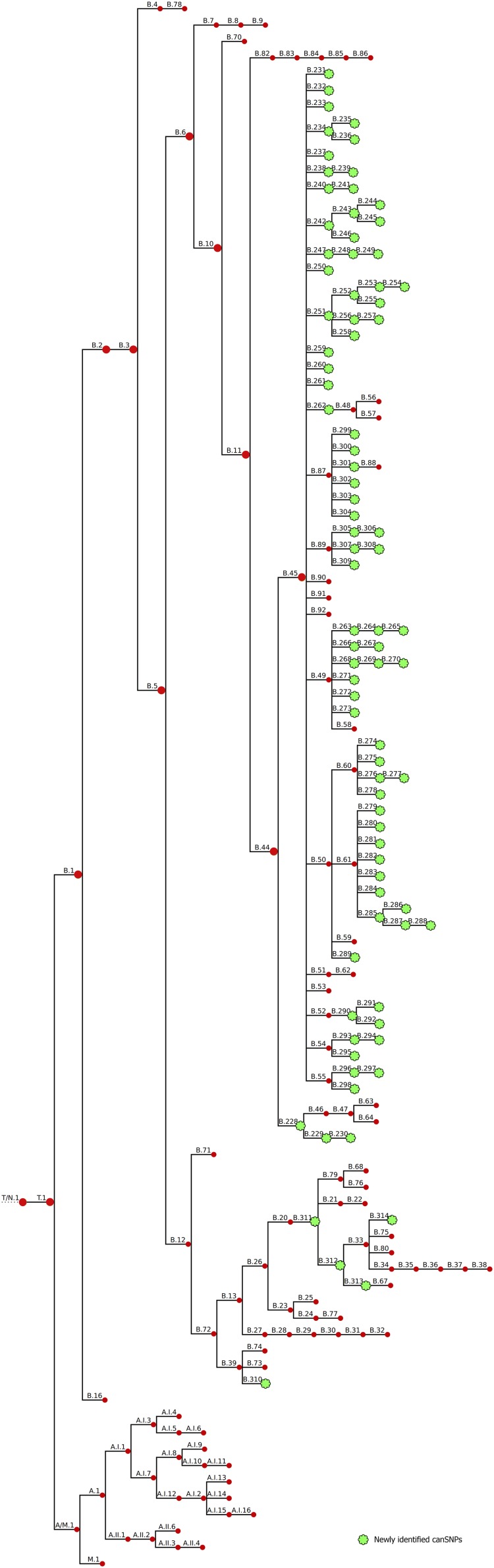
Revised CanSNP nomenclature. Newly identified clades are indicated in green.

To obtain a higher resolution power, a whole-genome SNP analysis was carried out. We accurately reconstructed the population structure according to the global phylogenetic framework (Supplementary Figure S1). Based on SNP analysis, clade B.6 displayed very low genetic diversity compared to clade B.12 (Supplementary Figure S3). All strains assigned to clade B.12 displayed the A to C nucleotide change at position 2059 of the *rrl* gene (*E. coli numbering*), the SNP associated with erythromycin resistance (Karlsson et al., 2016), and showed *in vitro* erythromycin resistance (data not shown). Four strains isolated from the South of France appeared to share genotypes with B.12 isolates, which is consistent with the CanSNPer typing results (i.e. subclades 13 and B.39). By comparing the phylogenetic tree of the 350 strains with the CanSNPer results, 87 new subclades were highlighted, among which 82 derived from clade B.44. On an alignment of 26,463 SNPs identified in our sample set, 87 canSNPs were therefore added to the canSNP nomenclature (Figure 2).

We were able to define five new canSNPs in the B.12 clade. One new subclade of clade B.39 was identified with six specific SNPs (B.310). Two intermediate canSNPs were also added: (i) subclade B.311, which was characterized by fifteen specific SNPs and one specific deletion, and (ii) subclade B.312, distinguished by ten specific SNPs, from which B.33 and the new subclade B.313 derive. One new subclade of B.33 was also identified (B.314, three specific SNPs).

In the previous canSNP nomenclature, clade B.44 – the major clade found in France – included two subclades: B.45 and B.46. Resolution was added to the branches deriving from B.44: a novel clade B.228 that still splits into clade B.46 but also into clade B.229 (that includes the B.230 subclade as well). These subclades were distinguished by one to three specific SNPs each. Clade B.45 formed a tight cluster composed of almost all strains from France. Seventy-nine new canSNPs were defined for this clade. Clade B.45 and all its subclades displayed a star phylogeny, i.e. “a multifurcation with many short branches connected at an internal node,” with an average of only one to three specific SNPs per branch. Most supported subclades were B.308 with nine specific variants (including one deletion) and B.298 with six specific variants (including two deletions). Seven other groups were well supported, with four to five specific SNPs each (B.234, B.244, B.261, B.272, B.291, B.294, and B.297 – bootstraps between 98 and 100). CanSNPs of clade B.44 were classified from D1 to D8 (corresponding to a gradual increase in discrimination) to facilitate investigation of the described clusters at different levels (Table 3). Newly identified canSNPs were implemented in the canSNP nomenclature and publicly available genomes were typed *in silico* (Supplementary Table S3). The following newly identified clades were detected among these publicly available genomes: B.228, B.251-254, B.260, B.262-B.265, B.269, B.279, B.284, B.285, B.288, B.290, B.291, B.295, B.301, B.305, and B.310-314. All newly identified canSNPs were confirmed as non-homoplastic, except for B.257 and B.288 that were also detected in two and one *F. novicida* strains, respectively (data not shown). However, as it does not concern *F. tularensis* species, these canSNPs were not excluded from the nomenclature.

**TABLE 3:** CanSNPs were classified between D1 and D8, which correspond to different orders of discrimination.

**D1**		**D2**		**D3**		**D4**		**D5**		**D6**		**D7**		**D8**	
B.4	*1*														
B.6	*319*	B.44	*317*	**B.228**	***5***	B.46	*2*	B.47	*1*	B.63	*1*				
										B.64	*0*				
						**B.229**	***3***	**B.230**	***2***						
				B.45	*312*	**B.231**	***4***								
						**B.232**	***2***								
						**B.233**	***4***								
						**B.234**	***4***	**B.235**	***2***						
								**B.236**	***2***						
						**B.237**	***4***								
						**B.238**	***4***	**B.239**	***2***						
						**B.240**	***5***	**B.241**	***2***						
						**B.242**	***7***	**B.243**	***5***	**B.244**	***2***				
										**B.245**	***2***				
								**B.246**	***2***						
						**B.247**	***7***	**B.248**	***6***	**B.249**	***2***				
						**B.250**	***10***								
						**B.251**	***15***	**B.252**	***6***	**B.253**	***3***	**B.254**	***2***		
										**B.255**	***3***				
								**B.256**	***5***	**B.257**	***2***				
								**B.258**	***2***						
						**B.259**	***3***								
						**B.260**	***2***								
						**B.261**	***2***								
						**B.262**	***7***	B.48	*2*	B.56	*2*				
										B.57	*0*				
						B.49	*44*	B.58	*0*						
								**B.263**	***7***	**B.264**	***6***	**B.265**	***2***		
								**B.266**	***5***	**B.267**	***2***				
								**B.268**	***5***	**B.269**	***3***	**B.270**	***2***		
								**B.271**	***4***						
								**B.272**	***2***						
								**B.273**	***2***						
						B.50	*65*	B.59	*3*						
								B.60	*15*	**B.274**	***2***				
										**B.275**	***2***				
										**B.276**	***3***	**B.277**	***2***		
										**B.278**	***2***				
								B.61	*36*	**B.279**	***3***				
										**B.280**	***2***				
										**B.281**	***3***				
										**B.282**	***2***				
										**B.283**	***4***				
										**B.284**	***2***				
										**B.285**	***10***	**B.286**	***3***		
												**B.287**	***6***	**B.288**	***3***
								**B.289**	***4***						
						B.51	*2*	B.62	*1*						
						B.52	*8*	**B.290**	***6***	**B.291**	***3***				
										**B.292**	***2***				
						B.53	*5*								
						B.54	*5*	**B.293**	***3***	**B.294**	***2***				
								**B.295**	***2***						
						B.55	*17*	**B.296**	***3***	**B.297**	***2***				
								**B.298**	***2***						
						B.87	*36*	**B.299**	***3***						
								**B.300**	***3***						
								**B.301**	***3***	B.88	*0*				
								**B.302**	***2***						
								**B.303**	***2***						
								**B.304**	***2***						
						B.89	*9*	**B.305**	***3***	**B.306**	***2***				
								**B.307**	***3***	**B.308**	***2***				
								**B.309**	***2***						
						B.90	*0*								
						B.91	*1*								
						B.92	*2*								
B.12	*27*	B.13	*24*	B.26	*23*	**B.311**	***20***	B.79	*0*						
								B.21	*4*	B.22	*2*				
								**B.312**	***16***	B.33	*13*	**B.314**	***2***		
												B.75	*0*		
												B.80	*2*		
												B.34	*7*		
										**B.313**	***2***	B.67	*0*		
						B.23	*3*	B.24	*1*	B.77	*1*				
				B.27	*0*										
		B.39	*4*	B.73	*2*										
				B.74	*0*										
				**B.310**	***2***										
B.16	*0*														

*The number of strains (n = 350) identified in each clade is indicated in italics. Newly identified subclades are indicated in bold.*

### Spatiotemporal Distribution of *F. tularensis*

**FIGURE 3:**
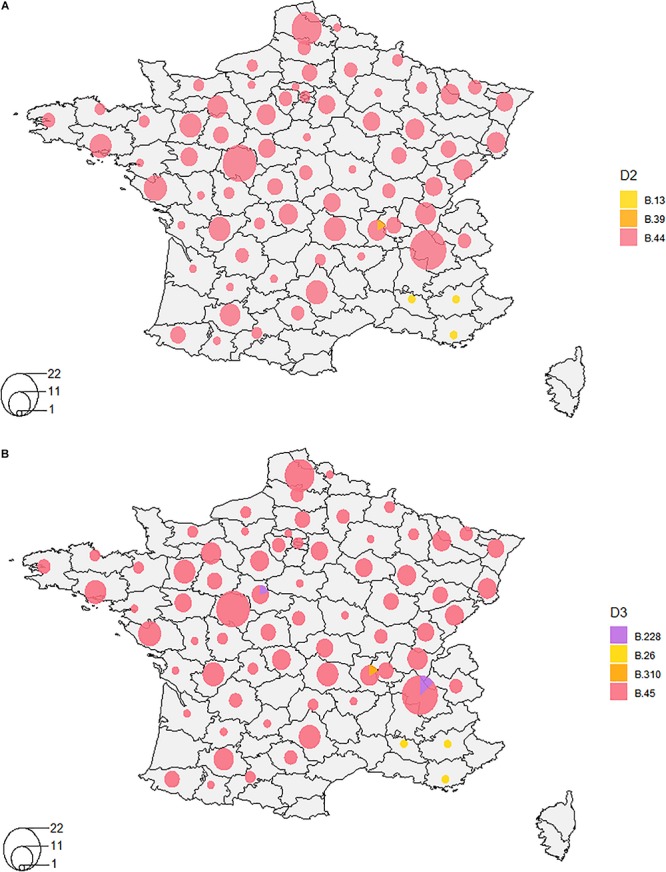
Distribution of clades in France on orders of discrimination D2 **(A)** and D3 **(B)**. A- Clade B.44 was the major clade identified in France on the order of discrimination D2, except for four strains isolated in the South-East of France that belonged to clade B.12 (B.13 and B.39). B- On the order of discrimination D3, clade B.45 is the major clade. Clades B.228, B.26, and B.310 were also detected to a lesser extent.

We were able to highlight the major characteristics of the geographic and temporal distribution of *F. tularensis*. The difference between biovars was well established, with clade B.12 (D1) detected in Germany and Sweden, and clade B.6 (D1) detected in France, Germany, Spain, Sweden and Belgium. More specifically, clade B.44 (D2) was the major clade identified in France (Figure 3A). Strains from France identified within clade B.12 (B.13 + B.39 – D2) were isolated only in the South East region of the country. Strains assigned to the newly identified clade B.228 (D3) were isolated mostly in the Alps region, near the Eastern border of France (Figure 3B). One B.230 strain (clade B.228–229) was isolated 30 years ago in the center of France, relatively far from the Alps region. Clade B.46 was identified in the Alps region only in the 2010s.

When increasing resolution, we were not able to highlight any distinct clustering depending on the geographic location of isolation (Supplementary Figure S1), except for clade B.243 (D4–1998 to 2014, *n* = 5), clade B.239 (D5–1993, *n* = 2), clade B.241 (D5–2006, *n* = 2), and clade B.244 (D6–1999, *n* = 2) that were specific to one French department each. However, we highlighted a significant association between some genotypes and their geographic distribution on the order of discrimination D4 (Fisher’s exact test, *p* = 0.0005, Table 4). Indeed, clade B.49 was mostly isolated in the East of France (57%), while clades B.50, B.55, B.87, and B.251 were mostly isolated in the Western region of France (47, 47, 86, and 67%, respectively) (Table 4). These clades were associated with the highest number of strains and subclades. However, strains isolated over long geographic or temporal distances could share the same clade. Clade B.52, for example, was identified for strains isolated in France and Spain, from 1988 to 2016 and in 2008, respectively. However, three Spanish strains could be discriminated on the 5th order of discrimination D5 (B.291, a subclade of B.52 defined by four SNPs). Also, one strain isolated from a hare in 2012 in Germany clustered in the order of discrimination D7 (clade B.287) with two strains isolated in 2008 from capuchin monkeys (*Cebus* sp.) in a zoo and three strains isolated from humans who contracted oropharyngeal tularemia in 2008. Clade B.288 (D8) allowed us to discriminate strains that correspond to one of the rare grouped cases of tularemia. This reflects the need for highly discriminative SNPs to investigate tularemia outbreaks. Among the eight strains isolated in the same department, only one isolated in 1995 belonged to the same D4 clade (B.50). However, the two strains isolated from the capuchin monkeys (*Cebus* sp.) were isolated in a neighboring department during the same year and could be linked to this grouped case.

**TABLE 4:** Distribution of strains assigned to clades B.49, B.50, B.55, B.87, B.250, and B.251 (D4 clades with more than 10 strains) depending on their geographic location in France.

	**No. of strains**	**West %**	**Center %**	**East %**
B.49	**40**	30	12.5	57.5
B.50	**57**	47.4	38.6	14
B.55	**17**	47.1	29.4	23.5
B.87	**35**	85.7	5.7	8.6
B.250	**10**	30	30	40
B.251	**15**	66.7	20	13.3

### Spatiotemporal Analysis

**FIGURE 4:**
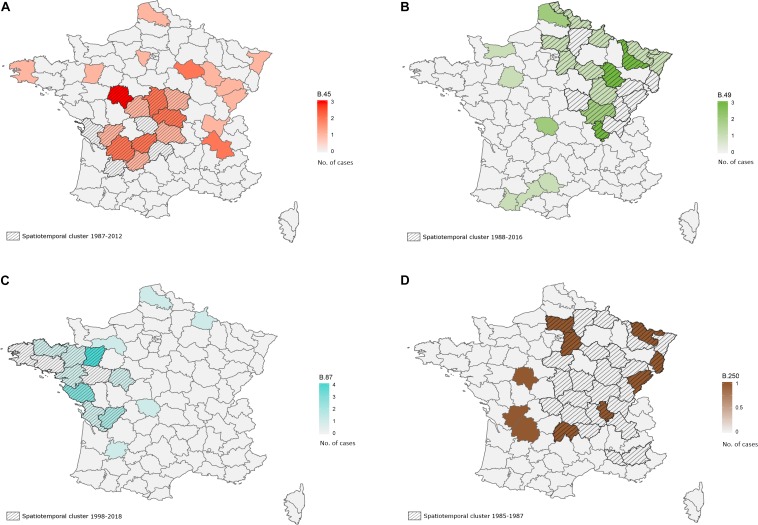
Spatiotemporal clusters detected in hares on order of discrimination D4 for clades B.45 (strains that are not discriminated after B.45, 1987–2012, **A**), B.49 (1988–2016, **B**), B.87 (1998–2018, **C**) and B.250 (1985–1987, **D**). French departments are colored with a gradient depending on the number of isolated strains belonging to the concerned clade. The identified spatiotemporal clusters are crosshatched.

A significant association between the geographic and genetic distance matrices was identified for strains isolated from hares between 1985 to 2018 in France (*n* = 204; Mantel’s test, *p* = 0.002). However, this association was not identified within clades (order of discrimination D4) containing at least 10 strains of our sample set (B.45, B.49, B.50, B.55, B.87, B.250). We identified spatiotemporal clusters for clade B.45 (strains not assigned to any subclade on the order of discrimination D4) and clades B.49, B.87 and B.250. A spatiotemporal cluster for clade B.45 (*n* = 36) was identified in the Center of France, including 15 strains isolated from 1987 to 2012 (Figure 4A, *p* = 0.02). Another spatiotemporal cluster was detected for 19 strains belonging to clade B.49 (*n* = 51) in the East of France between 1988 and 2016 (Figure 4B, *p* = 0.004). Finally, two other clusters were identified for clade B.87 (*n* = 36) with 14 strains isolated from 1998 to 2018 in the West of France (Figure 4C, *p* = 0.0003), and for clade B.250 (*n* = 10) with 7 strains isolated in the eastern half of the country between 1985 and 1987 (Figure 4D, *p* = 0.0001).

### Temporal Evolution of *F. tularensis* in France

**FIGURE 5:**
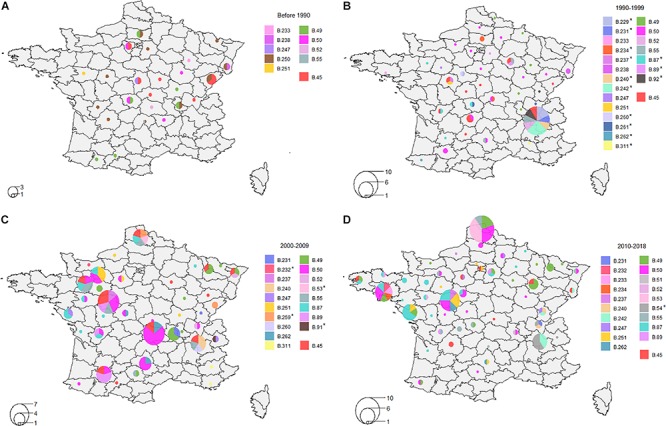
Distribution of clades on order of discrimination D4. **(A)** Distribution between 1947 and 1989; **(B)** Distribution between 1990 and 1999; **(C)** Distribution between 2000 and 2009; **(D)** Distribution between 2010 and 2018. Clades marked with a star indicate clades that were not present before this period.

Over the last four decades, tularemia seems to have spread from Eastern-Central France to the northern and western areas of the country (Figures 5A–D). Clades B.49, B.50, B.52, and B.55 (D4) were identified in strains isolated before 1990, and the new nomenclature enabled us to identify five other D4 clades (B.233, B.238, B.247, B.250, and B.251) that seemed to be part of the first genotypes observed in France (Figure 5A). Interestingly, strains assigned to clade B.45 on the 4th order of discrimination D4 (i.e. no canSNP detected after B.45) have been isolated since 1947 and were still isolated in 2018. Genotypes such as B.49, B.50, B.52, B.55, B.233, and B.247 (D4) were identified in strains isolated from non-neighboring French departments over a period of around 30 years (Figure 5). Clade B.251 also displayed strains isolated in non-neighboring departments but seems to have evolved mostly locally, from one French department (Maine-et-Loire) to neighboring departments in the north-western area (Figure 5). Similarly to B.251, clade B.49 seems to have spread to the East from 1986 to 2018. Clade B.50 was, however, largely distributed across France, and most of the strains belonged to this clade (Table 1). Interestingly, clades that seemed to persist in France were also those displaying the highest number not only of strains but also of derived subclades, testifying to the evolution of these clades. Some clades seem to have disappeared, such as clade B.250 that comprised ten strains isolated from 1985 to 1987 in ten different departments of France (Figure 5A), or clade B.238 that was isolated only from 1988 to 1996 (Figures 5A,B). Clades B.259, B.260, and B.261 were isolated in the past but were no longer detected in recent years (Figures 5B,C). Conversely, certain genotypes seem to have emerged over the last few decades (Figures 5B–D) – genotypes marked with a star. We can note that most of the clades were identified in strains isolated in the 1990s (Figure 5B). Some clades seem to be sporadic, as they were not identified in the last 10 (B.91 or B.259 - Figure 5C) to 20 years (B.92 - Figure 5B). Clade B.87 appeared in 1994 in the western areas and seemed to pursue its spread to the west and north (Figures 5B–D). Clades B.54, B.240, and B.231 seem to be restricted to the eastern region (Figures 5B–D). Interestingly, clade B.54 involved only human strains in our study. More recent genotypes have been identified, such as B.53 - formerly identified in Switzerland and Germany – that was isolated in 2007 near the northern border of France, and strains assigned to B.53 (*n* = 5) were strictly identical (no differences in SNPs nor INDELs). All of these results suggest that the genetic diversity of *Fth* strains is still evolving in France.

### Host Species

Strains used in this study were mostly isolated from hares, but also from humans or others mammals (captive or wild), with some animal cases directly linked to human cases. No clustering was observed as different host species could share identical genotypes (Supplementary Figure S1), and no association between genotypes and host species was detected (Fisher’s exact test, *p* > 0.05, data not shown). Therefore, there is no evidence of host specialization during the transmission of the bacterium. For example, genotype B.53 was shared between a European brown hare (*Lepus europaeus*) and the tick it was carrying (data not shown). Moreover, we observed that genotypes could differ among a given host species: two strains isolated during the same year in two roe deers (*Capreolus capreolus*) from the same department differed by 13 SNPs.

Concerning strains from the collection of the French National Reference Center for *Francisella*, genotypes were not associated with clinical forms (Supplementary Figure S2A). Also, there was no clinical form that was more often diagnosed depending on the year of isolation (Supplementary Figure S2B). Concerning antibiotic resistance, besides the erythromycin resistance observed in biovar II, only *blaFTU-1* (Accession: A7J11_01422), a gene coding for a class A beta lactamase, was detected in the studied strains. This gene is associated with betalactam resistance in *F. tularensis* (Antunes et al., 2012).

## Discussion

### Genetic Diversity in France

In 2011, only subclade B.Br.FTNF002-00 (Vogler et al., 2011) was identified as the major clade in France, reflecting low genetic diversity among *Fth* strains found in Western Europe, compared to those found in Eastern and Central Europe. Later, Dwibedi et al. (2016) identified B.45, B.49, B.60, B.54, and B.55 (clade B.44) as the only subclades present in France. We have now highlighted the presence of almost all B.44-subclades in France, except for B.57, B.58, B.62, B.64, B.88, and B.90. Along with the identification of 87 new subclades, among which 82 derive from clade B.44, these results illustrate for the first time some genetic diversity among *Fth* isolates from France. Moreover, like Vogler et al. (2011), we identified clade B.12 among very few French isolates (Vogler et al., 2011). The four strains identified in clade B.12 (mostly found in Central and Eastern Europe) were isolated in the South of France, where it is known that hares were imported from Eastern Europe for hunting purposes. It is highly likely that tularemia was introduced from Eastern Europe to Western Europe because of this practice (Olivier and Mondain-Monval, 2010). Indeed, hares have frequently been released in France over the past 30 years and particularly in the South of France, with 1 to 3% of the national hare population consisting of imported hares (Office National de la Chasse et de la Faune Sauvage). For this reason, Vogler et al. did not consider the B.12-strains as representative of an ecologically established population in the South of France, even though this region neighbors the Alps region, where both clades B.6 and B.12 are detected (Dwibedi et al., 2016). Strict recommendations concerning imports are now given for countries that are considered infected by *F. tularensis* (World Organization for Animal Health (OIE), 2014). Nowadays, although no official statistics are available, released hares mostly originate from farms and, to a lesser extent, from imports. Further epidemiological surveillance of tularemia in France will help to decipher whether clade B.12 is naturally present in France.

### Geographic Distribution

We confirmed previous phylogeographic studies showing that clade B.12 is present in Central and Eastern Europe, while clade B.6 is restricted to Western Europe, with these two clades overlapping in countries of Central Europe (Vogler et al., 2009; Gyuranecz et al., 2012). We also confirmed *in vitro* and *in silico* erythromycin resistance for strains assigned to clade B.12 and *in silico* betalactam-resistance for all strains. These findings were consistent with minimum inhibitory concentration (MIC) data obtained for strains at the French National Reference Center for *Francisella* (Caspar et al., 2018).

The overrepresentation of clade B.11, and more particularly clade B.44, suggests the introduction and spread of one clone of *Fth* across France. The star phylogeny observed for clade B.44 and all its subclades, along with the relatively low diversity observed among our strains, demonstrate that rapid clonal expansion of a monophyletic population of *F. tularensis* occurred in Western Europe. Isolation of strains assigned to clade B.46 was limited to the Alps region and to Germany. It has been suggested that clade B.46 had a longer evolutionary history as the genetic diversity in this region was higher, and that the presence of all the major clades (B.11, B.45, B.46, B.47) in Switzerland was evidence for an evolutionarily older founder population (Dwibedi et al., 2016). Accordingly, the two strains assigned to clade B.46 in our study were isolated from the French Alps region. Since synchronicity between epidemics and epizootics has been highlighted by epidemiologic data (Mörner, 1992), a study on pathogenicity could then be carried out in order to determine whether the low number of isolates is due to a lower number of animals infected by strains of this clade, or whether animals succumb more rapidly to tularemia, making it difficult to detect strains in this clade. Moreover, as our collection of animal strains depends on the SAGIR surveillance network for France, collection of animals might not be equivalent for all areas (Moinet et al., 2016). The possibility that tularemia is underdiagnosed should thus be considered, especially when tularemia is not targeted, as only dead or dying animals are collected. Also, some regions might be more prone to hunting or might not contain the same biodiversity. To limit this collection bias, strain selection was carried out in order to account for biodiversity in France. Besides, identification of new markers highlighted a novel intermediate node, B.228, from which B.229 and B.46 derive. Therefore, there is a common ancestor to these two clades and clade B.229 was not identified in any other country than France, according to the genomes publicly available so far.

We identified an association between clades (B.49, B.50, B.55, B.87, B.250, and B.251) and their geographic distribution. Interestingly, the fact that strains that belong to clade B.45, but that could not be discriminated at higher levels of resolution, formed a spatiotemporal cluster between 1987 and 2012 demonstrates that the bacterium is able to trigger outbreaks over long periods with a very slow mutation rate. Specifically, this spatiotemporal cluster was mostly located in an area of arable lands, where high densities of hares can be observed (Smith et al., 2005). We identified spatiotemporal clusters for clades B.49, B.87, and B.250 as well. Clade B.87 is relatively recent in France and seems to predominate in the West of France, according to the spatiotemporal cluster identified between 1998 and 2018, unlike clade B.49 for which a spatiotemporal cluster was identified between 1988 and 2016 in the East of France, testifying to its long-term presence on the French territory. Moreover, we identified a correlation between genetic distance and geographic distance in order of discrimination D4, which is consistent with the presence of these spatiotemporal clusters for the predominant clades. According to the concept of natural nidality of Pavlovsky (Pavlovsky, 1966), the bacterium could persist under defined conditions and trigger repeated outbreaks in certain locations, as proposed in Sweden by Johansson et al. (2014). However, inside genetic clades, we could not highlight any correlation between genetic distance and geographic distance. While a previous study highlighted this type of correlation for clade B.52 in Spain (Dwibedi et al., 2016), identification this clade in France in 1988 suggests dispersal of the bacterium from France to Spain as tularemia outbreaks in Spain have first been reported since 1997 (De Mateo and Ruiz Coisin, 1998; Allue et al., 2008; Ariza-Miguel et al., 2014). Moreover, the outbreak in 1997 was associated with hunting and handling of European brown hares (*L. europaeus*) in northwestern Spain, which seems to coincide with a spread from France to Spain.

Dispersal of *F. tularensis* through animals could be explained by hare import (Olivier and Mondain-Monval, 2010), as hares are mostly sedentary, or by migratory birds, in which tick-borne pathogens have been detected (Lopes de Carvalho et al., 2012). The role of birds in tularemia transmission was shown after a human case related to a buzzard (*Buteo buteo*) (Padeshki et al., 2010). Hematophagous arthropod vectors (i.e. flies and mosquitoes) also play an important role but, at least in France, this role in tularemia transmission has been poorly studied. A recent study in Switzerland, where ticks were found to be the main cause of infection in humans (Lyko and Chuard, 2013), suggested that strains of clade B.45 were better adapted than other clades to arthropods vectors, as clade B.46 and the newly identified B.11 subclades were isolated only in humans and mammals (Wittwer et al., 2018). However, in France, tick bites represent only around 10% of tularemia cases (Maurin et al., 2011) and Michelet et al. (2014) suggested that the risk of acquiring tularemia from ticks – *Ixodes ricinus* at least – was negligible. The nature of the epidemiological cycle (terrestrial or aquatic) remains to be elucidated. In Scandinavia, where the aquatic cycle predominates, mosquitoes represent the principal reservoir of tularemia and triggered significant outbreaks (Tärnvik, 2007). Also, recent studies have demonstrated the role of flower nectar in the *F. tularensis* lifecycle with mosquitoes as vectors (Kenney et al., 2017). In Norway, human outbreaks of water-borne tularemia have been linked with high population density of lemmings (*Lemmus lemmus*) and drinking water from wells contaminated by carcasses of infected lemmings (Berdal et al., 2000; Larssen et al., 2011). In France, for three human cases linked to water exposure in the same river between 2009 and 2014, water samples were analyzed but *Francisella* sp. Was not identified (Guerpillon et al., 2016). Therefore, further investigations need to be conducted on the persistence of *F. tularensis* in water, especially in France. Wind-assisted dispersal is also one of the hypotheses for the spread of *F. tularensis* across long distances. Otherwise, long-range dispersal of similar clones of *F. tularensis* could be related to the low pathogenicity observed among *Fth*. Finally, by infecting hosts without killing them, contrarily to *Ftt*, the bacterium would be able to spread more rapidly and over longer distances. In Europe, differences in disease characteristics in brown hares (*L. europaeus*) have been observed, with splenitis and hepatitis in hares infected with strains belonging to clade B.FTNF002-00, and polyserositis when infected with clade B.13 (subclade of B.12) (Kreizinger et al., 2016). Further investigations should be conducted on this difference of pathogenicity between clades of Western Europe and clades of Central and Eastern Europe.

### Temporal Evolution

As tularemia presents complex epidemiologic cycles that have not yet been elucidated (terrestrial and aquatic cycles, wide range of host species and reservoirs, possible transmission through vectors), it is likely that some biotopes or climatic conditions could influence components of these cycles and thus favor or hinder the spread and infection capacities of some clades. Clade B.250 illustrates this hypothesis as it was identified only between 1985 and 1987. The fact that a spatiotemporal cluster was identified for this clade (*p* = 0.0001) is consistent with this observation. We can therefore assume that the fitness of genotypes depends on specific conditions, such as cold waves that occurred in France in January 1985, February 1986, and January 1987. Climatic factors could affect either the bacterium, the host, or the vectors in terms of survival or activity. Such clades might have been adapted to a certain kind of climate, or there might have been a change in animal movements that led to the emergence and the disappearance of this clade in France. Also, in the 1980s, a significant decrease in the hare population in France was observed after the emergence of European brown hare syndrome (EBHS) (Marchandeau et al., 2011), which could explain the disappearance of these clades by lack of hosts. It is also conceivable that hunting might influence the hare population and therefore be responsible for the disappearance of some clades. Among many hypotheses explaining the disappearance of some clades, it is known that pesticides are responsible for considerable ecosystem changes and might modify animal habitats. It has also been shown that exposure to certain pesticides could potentiate the pathogenicity of tularemia in brown hares (Bandouchova et al., 2011), or pesticides could be more toxic to tularemia-infected voles (Vidal et al., 2009). Considering that hares most likely live around cultivated plains and cereal crops, the relationship between areas where pesticides are used (e.g. intensive farming areas) and tularemia could also be investigated.

The fact that identical genotypes could be identified over long geographic or temporal distances could suggest convergent evolution. However, as only non-homoplastic markers were selected, the existence of several groups sharing the same genotype in non-neighboring geographic areas, and over such long periods of time, supports the hypotheses of the ability of *F. tularensis* to persist in a quiescent stage and/or spread over long distances. The fact that clades that persist in France are associated with the highest number of strains and subclades also shows that some clades are more likely to thrive, but further investigations need to be conducted to assess why these clades are more able than others to survive and evolve. Some clade-specific SNPs were identified as non-synonymous variants, and it is known that synonymous SNPs might still play a role in the regulation of gene expression or the level of protein synthesis, even if they do not affect the protein sequence (Shabalina et al., 2013). However, 62% of the detected variants (including INDELs, data not shown) were responsible for silent mutations and it is difficult to assess whether the SNPs result from an adaptation process. Moreover, most of the identified genetic clades were supported by only one or a few variants. According to the neutral theory (Kimura, 1968), the vast majority of substitutions are unlikely to influence selective pressure, and evolution might rely on genetic drift at the molecular level. Genome-wide association studies will therefore be performed on the pan-genome in order to identify accessory genes (i.e. genes that are not shared by all strains) responsible for this distribution. When using such a large number of strains, we were able to observe that published markers resulted from variants that could be transient. This means that the persistence of the mutation over time is not yet proven, and that the concerned clade might disappear with natural selection, as some variants might not be fixed to the phylogenetic tree leaves. For example, a small group of six strains assigned to clade B.49 was not phylogenetically associated with the majority of clade B.49-strains, and one strain clustered with two other strains assigned to clade B.251 and B.45 (one order of discrimination less than B.49-Supplementary Figure S1). This shows that they shared more similarities phylogenetically between them than with other strains of their canSNP clade. As most canSNPs have been designed on final leaves of phylogenetic trees, selected SNPs are specific to these strains but actually do not allow us to place them accurately in a phylogenetic context. Therefore, for phylogenetic purposes, it is of great importance to select specific and robust SNPs (non-homoplastic SNPs with high bootstraps). However, as these canSNPs still clustered a large number of strains, we do not refute their importance in the canSNP nomenclature. Moreover, as canSNPs identified in our study were validated *in vitro*, and were well detected among publicly available genomes, low bootstrap canSNPs were kept as they could still provide information about some strains. Considering the ability of *F. tularensis* to persist with a slow replication rate and travel long distances over short periods of time, a clade that disappeared years ago in France could still re-emerge. Therefore, all subclade-specific SNPs were considered relevant and were implemented in the canSNP nomenclature. In order to further investigate the epidemiology of *F. tularensis* routinely and at lower cost, a high-resolution molecular typing tool is being designed in our laboratory for clades identified in France. This typing tool will be used according to the purpose of the study, with the appropriate order of discrimination: high level of resolution for outbreak investigation, or low level of resolution for long-term epidemiologic surveillance.

## Conclusion

In conclusion, we identified clade B.44 as the major clade present in France and identified 82 new subclades in this clade. The star phylogeny observed in clade B.44 subclades reveals that the biodiversity of *F. tularensis* in France is relatively recent and results from clonal expansion of a single population. We also highlighted specific features of the epidemiology of the bacterium, where *F. tularensis* is able to spread and thrive locally with a slow replication rate, but can also travel long distances since identical genotypes could be identified at large geographic but also temporal distances.

## Data Availability Statement

The project has been deposited at DDBJ/EMBL/GenBank under the BioProject number PRJNA551589 (https://www.ncbi.nlm.nih.gov/bioproject/PRJNA551589). Accession numbers (VIYS00000000–VJMD00000000) are available in Supplementary Table S1.

## Ethics Statement

The *F. tularensis* strain collection used in this study has been declared to and approved by the Agence National de Sécurité des Médicaments et des produits de santé (ANSM, Saint Denis, France) under authorization number ADE-067282015-5. Genetic material was used under authorization number AMO-076292016-8.

## Author Contributions

CP, NM, MM, and GG conceived the study and obtained funding. NM, MC, CM, MM, YC, HT, DG-W, and RE collected and maintained the biological materials as well as the associated data. MK, MC, CM, and YC performed DNA extractions. MK generated genomic data and performed data analysis. MK, GG, YC, and BD participated in data interpretation. BD performed statistical analyses. MK wrote the manuscript and GG, MM, YC, CP, and BD revised it critically. All authors contributed to manuscript revision, read and approved the submitted version.

## Conflict of Interest

The authors declare that the research was conducted in the absence of any commercial or financial relationships that could be construed as a potential conflict of interest.

## Footnotes

1 http://www.bioinformatics.babraham.ac.uk/projects/fastqc

2 ftp://ftp.ncbi.nlm.nih.gov/genomes/genbank/bacteria/Anaplasma_phagocytophilum/assembly_summary.txt

3 https://www.satscan.org/

## Supplementary Material

FIGURE S1 Maximum likelihood tree based on the core genome (SNPs) identified in the 350 sequenced strains. The tree was generated by IQtree following the TVM model (1000 replicates, SH-aLRT, UFBOOT2). Strains are associated with metadata (in order: country, host species, genetic clades (D4-D8), year of isolation, French administrative regions, clinical form when isolated from human). Colors of the branches, country of isolation and host species are colorized depending on the genetic clades (D4). Year of isolation is colorized in yellow when isolated before 2001 and in red when isolated between 2001 and 2018. French administrative regions and clinical forms are randomly colorized to be deciphered. Branch lengths are not used on the left figure to visualize phylogenetic relationships between strains. The right figure shows the phylogenetic tree using branch lengths.

FIGURE S2 Heatmap representing the genetic distance between each pair of strains (SNP matrix). Phylogenetic tree is associated with colors corresponding to genetic clades (D4).

FIGURE S3 Reported clinical forms. 1- Distribution of the genetic clades depending on the clinical form; B- Distribution of the reported clinical forms depending on the year of isolation. There was no evidence of an association between genetic clades and the reported clinical forms, nor between the year of isolation and the reported clinical forms.

TABLE S1 Metadata of the analyzed genomes with BioSample Accession, Genome Name, BioProject number, Accession, Major clade, Genetic clade, Isolation Date, Isolation Country, French department when available, Host Name, Deep coverage and Breadth coverage.

TABLE S2 List of the currently available and newly identified canSNP with the associated Genetic clade, Discrimination order, number of strains identified in the study, first and last year of isolation in the study, reference of the canSNP, reference genome and the state of the canSNP.

TABLE S3 CanSNPs associated with available genomes on https://www.ncbi.nlm.nih.gov/genome/genomes/511.

## Abbreviations

canSNP: canonical single nucleotide polymorphism
DNA: Deoxyribonucleic acid
*Fth, F. tularensis* subsp.: *holarctica*
*Ftt*: *F. tularensis subsp. tularensis*
INDELs: Insertions-deletions
ONCFS: *Office National de la Chasse et de la Faune Sauvage* (National Hunting and Wildlife Agency)
PCR: polymerase chain reaction
SNP: single nucleotide polymorphism.
