# Supplementary material for: Phylogeography and Genetic Diversity of Francisella tularensis subsp. holarctica in France (1947–2018)
Source: Front Microbiol. 2020 Mar 4;11:287. doi: 10.3389/fmicb.2020.00287 (PMC7064806; doi:10.3389/fmicb.2020.00287)
Supplement: FIGURE S1 — Maximum likelihood tree based on the core genome (SNPs) identified in the 350 sequenced strains. The tree was generated by IQtree following the TVM model (1000 replicates, SH-aLRT, UFBOOT2). Strains are associated with metadata (in order: country, host species, genetic clades (D4-D8), year of isolation, French administrative regions, clinical form when isolated from human). Colors of the branches, country of isolation and host species are colorized depending on the genetic clades (D4). Year of isolation is colorized in yellow when isolated before 2001 and in red when isolated between 2001 and 2018. French administrative regions and clinical forms are randomly colorized to be deciphered. Branch lengths are not used on the left figure to visualize phylogenetic relationships between strains. The right figure shows the phylogenetic tree using branch lengths. [file Image_1.pdf]

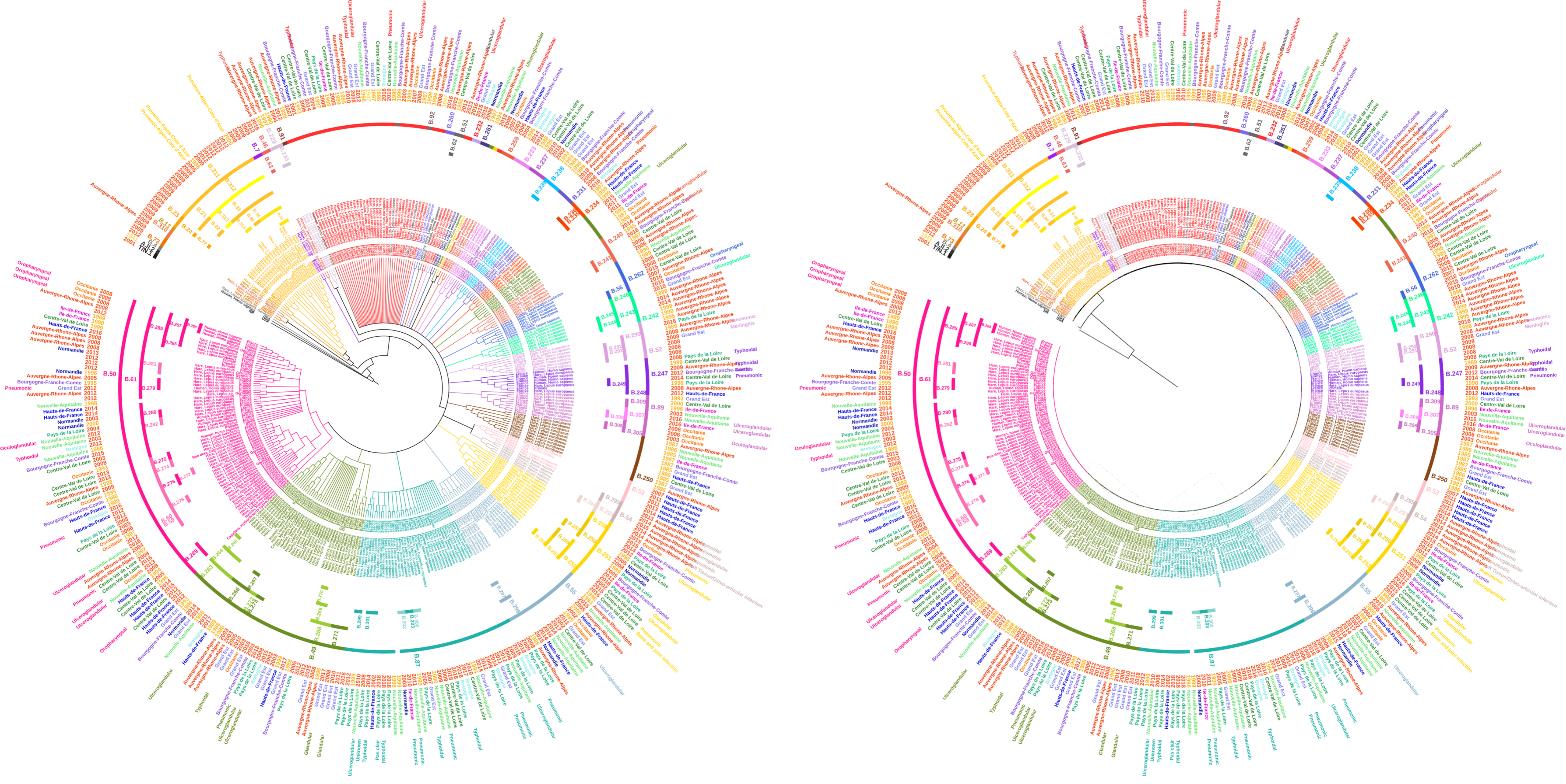

Figure S1. Maximum likelihood tree based on the core genome (SNPs) identified in the 350 sequenced strains. The tree was generated by IQtree following the TVM model (1000 replicates, SH-aLRT, UFBOOT2). Strains are associated with metadata (in order: country, host species, genetic clades (D4-D8), year of isolation, French administrative regions, clinical form when isolated from human). Colors of the branches, country of isolation and host species are colorized depending on the genetic clades (D4). Year of isolation is colorized in yellow when isolated before 2001 and in red when isolated between 2001 and 2018. French administrative regions and clinical forms are randomly colorized to be deciphered. Branch lengths are not used on the left figure to visualize phylogenetic relationships between strains. The right figure shows the phylogenetic tree using branch lengths.
