# Supplementary figures and images for: Phylogeography and Genetic Diversity of Francisella tularensis subsp. holarctica in France (1947–2018)
Source: Front Microbiol. 2020 Mar 4;11:287. doi: 10.3389/fmicb.2020.00287 (PMC7064806; doi:10.3389/fmicb.2020.00287)

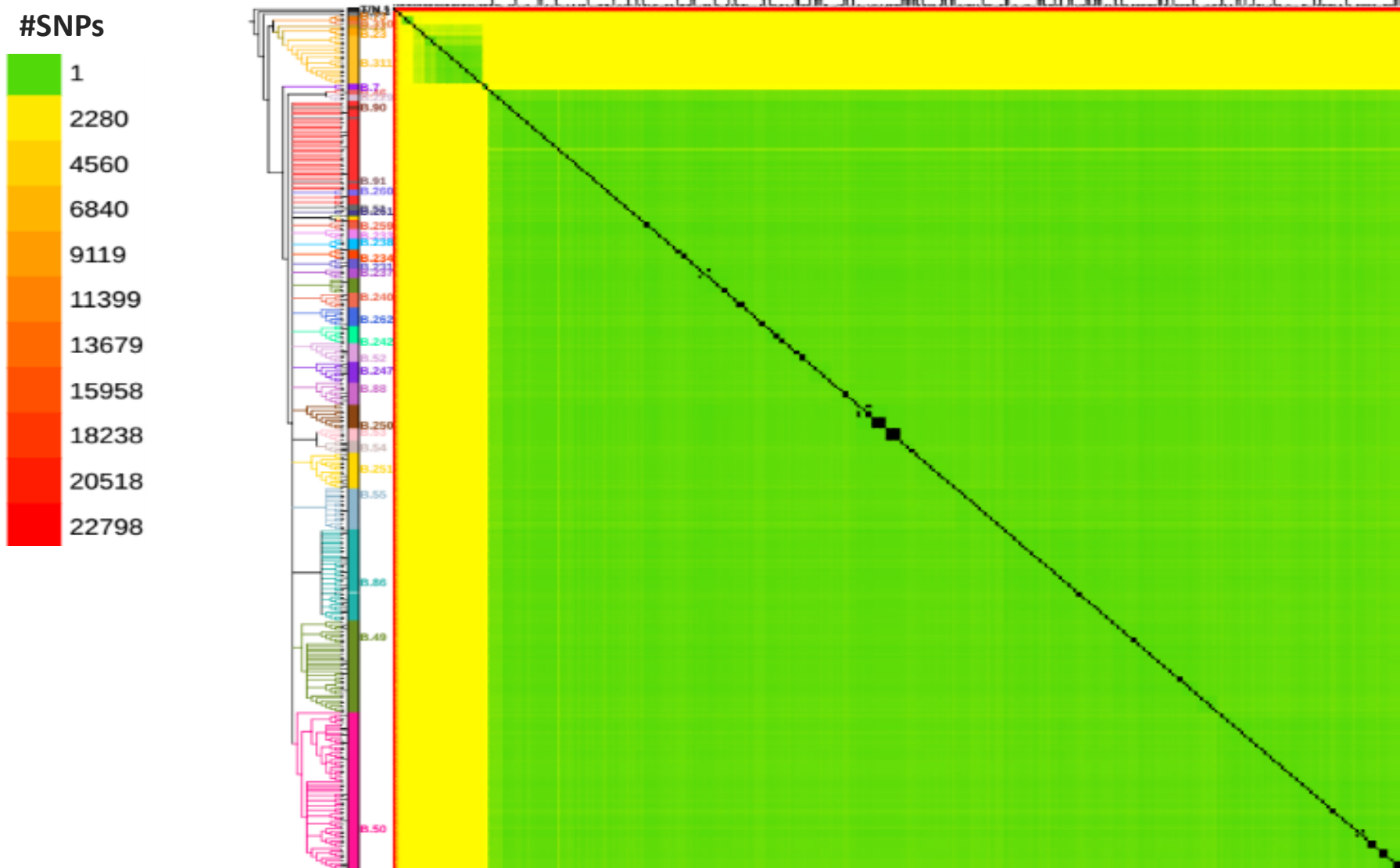

Supplement: FIGURE S2 — Heatmap representing the genetic distance between each pair of strains (SNP matrix). Phylogenetic tree is associated with colors corresponding to genetic clades (D4). [file Image_2.pdf]

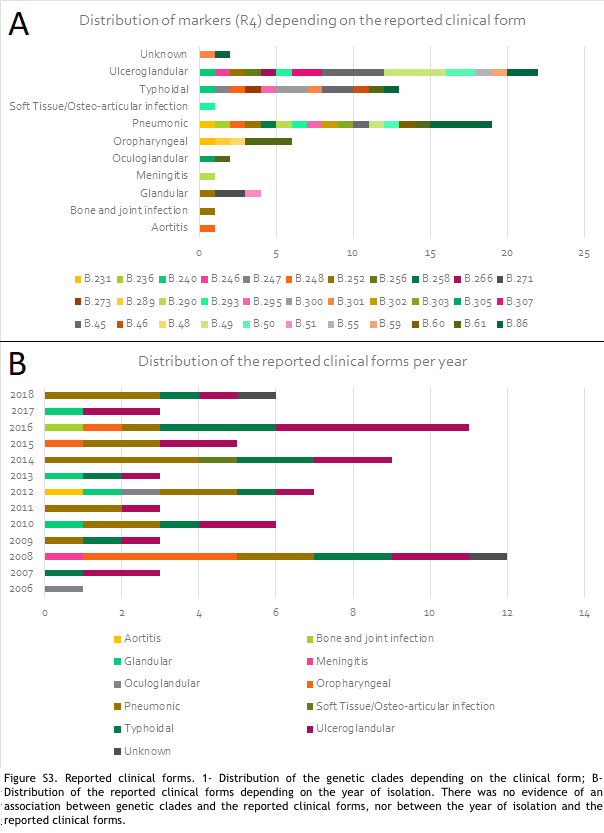

Supplement: FIGURE S3 — Reported clinical forms. 1- Distribution of the genetic clades depending on the clinical form; B- Distribution of the reported clinical forms depending on the year of isolation. There was no evidence of an association between genetic clades and the reported clinical forms, nor between the year of isolation and the reported clinical forms. [file Image_3.tif]
